# *Firmicutes*, *Bacteroidetes* and *Actinobacteria* in Human Milk and Maternal Adiposity

**DOI:** 10.3390/nu14142887

**Published:** 2022-07-14

**Authors:** Martha Alejandra Chavoya-Guardado, Edgar Manuel Vasquez-Garibay, Sandra Luz Ruiz-Quezada, María Inés Ramírez-Cordero, Alfredo Larrosa-Haro, Jorge Castro-Albarran

**Affiliations:** 1 Instituto de Nutrición Humana, Centro Universitario de Ciencias de la Salud, Universidad de Guadalajara, Salvador Quevedo y Zubieta #750, Edificio Anexo al Hospital Civil Dr. Juan I. Menchaca, Guadalajara 44340, Jalisco, Mexico; nutriologaalechavoya@gmail.com (M.A.C.-G.); alfredo.larrosa@academicos.udg.mx (A.L.-H.); 2 Laboratorio de Biología Molecular del Centro Universitario de Ciencias Exactas e Ingenierías, Universidad de Guadalajara, Blvd. Marcelino García Barragán #1421, Guadalajara 44430, Jalisco, Mexico; sandra.ruiz@academicos.udg.mx (S.L.R.-Q.); ines.rmzco@gmail.com (M.I.R.-C.); 3 Departamento de Ciencias de la Salud y Ecología Humana en el Centro Universitario de la Costa Sur, Universidad de Guadalajara, Av. Independencia Nacional #151, Autlan 48900, Jalisco, Mexico; nutjca@gmail.com

**Keywords:** *Firmicutes*, *Bacteroidetes*, *Actinobacteria*, human milk, maternal adiposity

## Abstract

The main objective was to explore the relationship between the microbiota of human milk and adiposity in Mexican mothers during the first lactation stage. Methods: Seventy lactating women were included. Adiposity by anthropometric measurements and by bioelectric impedance was obtained. The donation of human milk was requested, from which bacterial DNA was extracted and qPCR of the 16S region was performed. The Mann–Whitney U test, Spearman and Pearson correlations, and multiple linear regressions models were also calculated. Results: The median percentage of *Bacteroidetes* had a direct and significant correlation with normal adiposity, current BMI, waist circumference, and body fat percentage. The correlation with current BMI became significantly inverse in women with BMI ≥ 25. In women with normal BMI, the percentage of *Actinobacteria* showed a direct and significant correlation with current BMI, waist circumference, and percentage of body fat. Multiple linear regressions showed that pre-pregnancy BMI was the variable with the highest predictive value with the *Bacteroidetes* phyla in normal BMI and in BMI ≥ 25. Conclusions: the adiposity of the woman before pregnancy and during lactation would have an important effect on the abundance of *Bacteroidetes* and *Actinobacteria* in human milk.

## 1. Introduction

The intestinal microbiota in healthy adults reaches populations larger than 10^13^ microorganisms and it is considered an “organ” adjusted to our physiology [1]. It performs important metabolic and immunological functions and its development during childhood is considered crucial [2]. Human milk is considered the most important postnatal source of commensal bacteria to colonize the intestine of infants [3]. Under physiological conditions, bacterial concentrations in milk can be from 4log_10_ colony forming units (CFU)/mL [4]. To date, more than 303 different genera have been described, including *Bifidobacterium, Lactobacillus*, *Staphylococcus*, *Bacteroides*, *Enterococcus*, and *Streptococcus*, with a predominant abundance of the phyla *Firmicutes*, *Actinobacteria*, *Bacteroidetes*, and *Proteobacteria* [5].

Results in the past years suggest that certain bacteria from the maternal gastrointestinal tract could translocate to the mammary glands and subsequently colonize the gastrointestinal tract of the breast-fed neonate [6,7]. Direct correlations between the maternal fecal microbiota and the fecal microbiota of breastfed infants indicate that bacteria from the maternal intestine can access the mammary gland through the enteromammary pathway [8,9]. This is a process of bacterial translocation by which certain bacteria use phagocytic dendritic cells to penetrate the intestinal epithelium and travel through the circulatory system until they reach the mammary gland, colonize the milk, and later establish themselves in the intestine of the infant [10,11]. Thus, changes in the abundance and diversity of maternal enteric bacteria would be transferred to the intestine of the infant and would play a key role in the establishment of the infant’s intestinal microbiome [12,13,14].

It has been pointed out that maternal overweight and obesity are risk factors for the development of chronic diseases in offspring [15,16,17], and, although the mechanisms for this increase in risk are poorly understood, the transmission of the maternal microbiota through milk could play an important role [18,19]. A comprehensive characterization of the milk microbiota and knowledge of the factors that influence it would allow for a better understanding of its impact on the development of the intestinal microbiota of the infant and its health in the short and long term [20]. However, the potential transmission of bacteria that are part of the maternal intestinal microbiota through human milk to infants has been less studied. In particular, we are interested in exploring the relationship between the microbiota of human milk and adiposity in mothers during the first lactation stage.

## 2. Materials and Methods

A study was carried out with an analytical cross-sectional design nested in the longitudinal project “Impact of nonalcoholic beer on the microbiota of breast milk and its possible beneficial effects on the health of mothers and infants” (unpublished data). For this study, a call was launched on social networks to invite lactating women between 17 and 40 years old, apparently healthy, and with more than 30 and less than 180 days postpartum to donate human milk for scientific research. Those who had a full-term pregnancy (≥37 weeks), without having received antibiotics or antimicrobials a month before taking the sample and who did not report smoking, addiction, or abuse of alcohol and/or drugs were included.

Monthly home visits were scheduled with the mothers who met the inclusion criteria and showed an interest in participating. During the first visit, they were adequately informed about the study and the procedures to be carried out. During the visit, the mother’s general and clinical data were collected and anthropometric measurements, estimation of anthropometric indicators, and an evaluation of body composition by bioelectrical impedance analysis were obtained; everything was recorded in a specific format. If the mother did not have a suitable breast pump, she was given one free of charge (Harmony Manual Breast Pump by Medela, Baar, Switzerland). During a morning visit, the mother was instructed on the correct techniques for hand washing, sterilization, and proper use of the breast pump. A special collection bag for human milk (Medela) was provided and time was given to express milk from both breasts. For the present investigation, maternal information and an ounce of this human milk donated by each of the 70 women included were used.

Weight measurement was performed with the Tanita model TBF-310GS (Tokyo, Japan) without shoes and with the least number of garments possible. Height was determined with the SECA 213 stadiometer (Hamburg, Germany), with the participant barefoot, the position of the head in such a way that the auditory meatus and the lower edge of the eye socket were in a horizontal plane, and with the arms relaxed and on the back to the vertical stem. The BMI was calculated by dividing the weight of the person in kg by their height in meters squared (kg/m^2^). For the present study, a BMI < 18.5 was considered underweight, normal weight was a BMI 18.5–24.9, overweight was a BMI of 25.0–29.9, and obesity a BMI ≥ 30.0 [21].

For the waist circumferences, a SECA 201 anthropometric tape (Hamburg, Germany), was used. The waist circumference was taken in the narrowest place between the edge of the lower costal (10th rib) and the iliac crest [22]. The cutoff values to determine the risk of comorbidities were no cardiovascular risk equal to or less than 80 cm and a cardiovascular risk greater than 80 cm [21].

Pregestational weight and weight gain during pregnancy was self-reported by mothers. Weight gain during pregnancy was measured in kg and was categorized according to the recommendations of the Institute of Medicine (IOM) [23], where, according to the prepregnancy BMI, it was considered low weight gain when it was below the recommendations and adequate when it was within the limits. An adequate weight gain for underweight BMI was between 12.5 and 18 kg, normal between 11.5 and 16 kg, overweight between seven and 11.5 kg, and obesity between five and nine kg.

The analysis of body composition by bioelectric impedance analysis (BIA) was performed with a Tanita model TBF-310GS brand (Tokyo, Japan). The participant had an empty bladder and a fasting state of at least four hours. She had not exercised prior to the study, nor had she consumed alcohol the day before. Before taking the measurement, the equipment was programmed by entering sex and height, using the standard value for body type, and always subtracting 600 g from the body weight to reduce clothing bias. For the purposes of the present study, lean mass and fat mass were measured in kg and as percentages. For the percentage of total body fat the following categories were used, normal: ≤33%, high: 34–39%, and excessively high >39% [24]. In addition to the BIA, the percentage of total body fat was calculated by anthropometry using the Siri equation [24], for which the tricipital skinfold (TSF), subscapular skinfold (SSF), bicipital skinfold (BSF), and suprailiac skinfold (SISF) were used, which were measured in mm with the Lange Skinfold caliper [25].

The milk samples were transferred along a cold chain to the Molecular Biology Laboratory of the University Center for Exact Sciences and Engineering (CUCEI) of the University of Guadalajara, where they were stored at −20 °C. At the conclusion of the sample collection, the extraction of bacterial DNA was performed from the milk samples using the commercial ZymoBIOMICSTM DNA Miniprep kit (D4304) from Zymo Research Corp. (Irvine, CA, USA). The quantification in ng/µL and the purity ratio 260/280 were taken into account. qPCR analysis was performed with runs of 72 samples on the Rotor Gene kit (QIAGEN) with SYBR Green (Thermo Fischer Scientific, Waltham, MA, USA). The qPCR conditions were as follows: hold temperature of 95 °C for 10 min, followed by 30 cycles with a denaturation temperature of 95 °C for 15 s and an extension temperature of 60 °C for 45 s, ending with a curve melt.

The primers used were 926F (AAA CTC AAA KGA ATT GAC GG) and 1062R (CTC ACR RCA CGA GCT GAC) for total bacteria at a concentration of 0.15 µM; for the *Bacteroidetes* phylum: 798cfbF (CRA ACA GGA TTA GAT ACC CT) and cfb967R (GGT AAG GTT CCT CGC GT AT), using a concentration of 0.3 µM; for *Firmicutes*: 928F-firm (TGA AAC TYA AGG AAT TGA CG) and 1040FirmR (ACC ATG CAC CAC CTG TC), at a concentration of 0.25 µM; for the *Actinobacteria* phylum: Act920F3 (TAC GGC CGC AAG GCT A) and Act1200R (TCR TCC CCA CCT TCC TCC G), with a concentration of 0.4 µM [26]. These primers were previously verified in the primer-BLAST database of the National Center for Biotechnology Information (NCBI), obtained on 27 May 2019.

20 µL microtubes were used, with 10 µL of SYBR Green, 4 µL of DNA from the samples, and primers F and R according to the following amounts and concentrations. For total bacteria: 0.6 µL (concentration 0.15 µM); for *Bacteroidetes:* 1.2 µL (concentration 0.3 µM); for *Firmicutes:* 1 µL (concentration 0.25 µM); for *Actinobacteria:* 1.6 µL (concentration 0.4 µM). Purified water free of DNA and RNA was added. A positive control was included in each run from DNA extracted from healthy adult feces and a blank (purified water free of DNA and RNA) was added for each bacterial phylum. The calculation of the percentage of phyla was carried out by interpolating the results of the curve of each bacterial phylum using a determined threshold of 0.05 according to two standard curves made with a DNA mix of 10 samples of human milk and a known standard for *Firmicutes* and *Bacteroidetes* (The ZymoBIOMICS^®^ Microbial Community Standard II (Log Distribution) Catalog # D6310).

The capture of the database and the statistical analyses were carried out with the IBM-SPSS Statistics version 21 program. Descriptive statistics were performed on the quantitative and qualitative variables, expressed as the median, interquartile range, minimum and maximum, and as percentages and frequencies, respectively. The normality of the data was tested using the Kolmogorov–Smirnov test. Chi-square and Kruskal–Wallis tests were performed to explore the qualitative variables with the proportions of bacterial phyla. Since most of the data showed distributions that did not meet the normality criterion, the Mann–Whitney U test was used to compare the medians between the groups. Spearman and Pearson tests were used to verify the correlation between the indicators of maternal adiposity and the percentage of bacterial phyla. Finally, multiple linear regressions were performed to show the value of the maternal anthropometric variables that explained the variability on the percentage of human milk bacterial phyla. A *p* ≤ 0.05 was considered significant value.

Ethical considerations. The project did not put the participants at risk and adhered to the guidelines of the Declaration of Helsinki, 2013. The protocol was reviewed and approved on 11 December 2018 by the Ethics Committee: “Ethics in Research and Biosafety” of the University Center for Health Sciences of the University of Guadalajara with opinion number CI-07118. In this research, we only worked with microorganisms from risk Group I according to the classification of the “Regulation of the General Health Law on Research for Health” in laboratories that have adequate facilities, equipment, and documentation that comply with the technical standards according to the Official Mexican Standard NOM-087-ECOL-2002.

## 3. Results

### 3.1. Quantitative and Qualitative Characteristics of the Mother–Child Dyad

The age of the mothers who donated the milk samples included in this study ranged from 17 to 40 years, with a median of 30. Most were professionals with a full bachelor’s degree education. The infants were between 4 and 18 weeks old, 63% were born by cesarean section, the majority were male (64.3%), and exclusive breastfeeding predominated over partial breastfeeding as a feeding method (71.4% vs. 28.6%) (Supplementary Tables S1 and S2).

### 3.2. Maternal Anthropometric Characteristics and Bacterial Phyla in the Human Milk

Maternal anthropometric measurements and percentages of bacterial phyla are described with medians and interquartile ranges (IQRs) in Table 1 and Table 2. Most of the women who were part of this sample began their pregnancy with a normal prepregnancy BMI (47.1%). Overweight was the second most frequent (30%), and only one participant had a BMI below 18.5 kg/m^2^. At the time of anthropometry, normal BMI continued to be the most frequent (51.4%). In 34.3%, the women had a pregnancy weight gain higher than that recommended by the IOM, 2009 [23]. In 41.4%, the participants had a normal body fat percentage, 35.7% were high, and 22.9% were excessively high.

### 3.3. Comparison of the Medians of the Percentages of Bacterial Phyla in Human Milk According to the Maternal Anthropometric Indices and Indicators

Differences were observed between the medians of bacterial phyla when segmenting the maternal anthropometric data between normal vs. overweight and obesity, which, despite not being significant, were interesting. The milk of women with pregestational BMI ≥ 25, waist circumference above 80 cm, and body fat percentage ≥33% had higher percentages of *Actinobacteria* than the milk of women with these anthropometric variables within normal limits. The milk of women with a current BMI ≤ 24.9 had a lower median *Firmicutes* than women with a current BMI ≥ 25. Furthermore, it had a higher median *Bacteroidetes* (5.9% vs. 3.7%) and a lower *Firmicutes/Bacteroidetes* ratio (F/B ratio) (1.4 vs. 2.4). Comparison of the medians of bacterial phyla of human milk according to skinfolds, when divided into groups according to ≤1 standard deviation (SD) and >1 SD, did not show significant differences. Stratification according to prepregnancy BMI (≤29.9 and ≥30) showed that the percentage of *Bacteroidetes* was significantly higher in the milk of women with a low prepregnancy BMI (*p* = 0.047). Likewise, BMI also showed a higher percentage of *Bacteroidetes* with a current low BMI (*p* = 0.042).

### 3.4. Correlations and Explained Values between Bacterial Phyla of Human Milk and Maternal Anthropometric Variables

A correlation analysis was carried out between the bacterial phyla of human milk and the maternal anthropometric variables. We also analyzed these correlations by segmenting the sample according to the cutoff points of normal adiposity vs. high and very high adiposity. The *Firmicutes* phylum showed an inverse correlation trend with weight gain during pregnancy, BSF, and SISF (*p* < 0.2). The F/B ratio showed a direct trend with weight gain during pregnancy. When only women with normal adiposity were included, the F/B ratio showed a trend toward an inverse correlation with prepregnancy BMI and with weight gain during pregnancy when it was within the parameters of the gain of weight recommended by the IOM, 2009 [23].

When including all the women participating in this study, the maternal anthropometric variables showed a no significant direct trend between the percentage of *Actinobacteria* and weight, prepregnancy BMI, current BMI, waist circumference, percentage of body fat, weight gain during pregnancy, TSF, SSF, and SISF.

**Figure 1:**
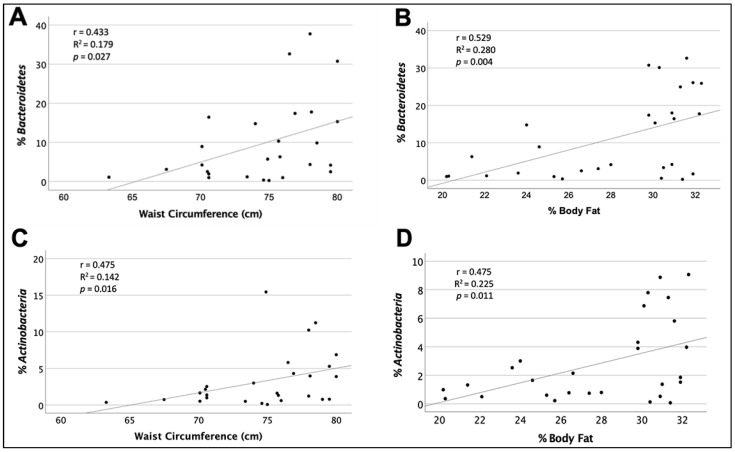
Correlation of the percentage of *Bacteroidetes* and *Actinobacteria* with waist circumference ≤80 cm and percentage of body fat ≤33%. (**A**) Regression between maternal waist circumference ≤80 cm and the percentage of the *Bacteroidetes* phylum in human milk; (**B**) Regression between the percentage of maternal body fat ≤33% and the percentage of the *Bacteroidetes* phylum in human milk; (**C**) Regression between maternal waist circumference ≤80 cm and the percentage of the *Actinobacteria* phylum in human milk; (**D**) Regression between the percentage of maternal body fat ≤33% and the percentage of the phylum *Actinobacteria* in human milk.

The indicators of adiposity that better explained the variability of the percentage of *Bacteroidetes* were, inversely, prepregnancy BMI ≥ 25 (16.2%) and, all directly, current BMI ≤ 24.9 (14.1%), waist circumference ≤80 cm (18.7%), fat percentage ≤33% (28.0%), BSF >1 SD (48%), and SISF >1 SD (79.7%). The highest direct abundance of the percentage of *Actinobacteria* were explained by a current BMI ≤ 24.9 (11.8%), waist circumference ≤80 cm (20.9%), fat percentage ≤33% (22.6%), and BSF >1 SD (67%), Table 3, Figure 1.

It is interesting how the *Bacteroidetes* and *Actinobacteria* phyla in human milk correlated directly with maternal abdominal adiposity when it was within healthy parameters, but when adiposity was higher this correlation showed an inverse trend between *Bacteroidetes* and waist circumference. In contrast, the adiposity located in the upper part of the body, represented by the bicipital and subscapular skinfolds, presented a significant direct correlation with both phyla when the skinfolds were >1 SD.

### 3.5. Multiple Linear Regressions (Stepwise Method) Were Performed with the Anthropometric Variables That Showed Important Correlations with the Percentages of Bacterial Phyla

Anthropometric variables were segmented according to the degree of adiposity. Prepregnancy BMI was the variable with the better explained value, showing significant results with the *Bacteroidetes* phyla both in BMI ≤ 24.9 (*p* = 0.011, adjusted R^2^ = 0.255) and in BMI ≥ 25 (*p* = 0.047, adjusted R^2^ = 0.114).

## 4. Discussion

The percentages of the bacterial phyla in human milk showed quite wide interquartile ranges. Despite the fact that our study included only mature milk and all of the samples were obtained in the same geographic region, a wide range of distributions was found among the proportions of the phyla studied. This great individual variability has already been described and, although it has been little studied, it has been associated with factors like geographical region, the stage of lactation, and the route of birth [1,26,27]. This study represents a window of opportunity to achieve a better understanding of the human milk microbiota.

The percentage of *Bacteroidetes* was directly correlated with the prepregnancy BMI, current BMI, waist circumference, and percentage of body fat when these indicators of adiposity were within the limits considered normal. However, this correlation was inverse when the cutoff points considered healthy were exceeded. These findings are similar to those observed by other authors for the intestinal microbiota [26,28,29], who described a higher proportion of *Bacteroidetes* in the human intestine associated with a normal BMI and a decrease in this phylum with a higher BMI.

Both BMI at the time of the study and prepregnancy BMI showed significant correlations with the percentage of *Bacteroidetes*, directly when BMI was in ranges considered healthy and inversely when BMI was greater than 25 points. These findings could mean that the abundance of this phylum in human milk is influenced by maternal nutritional status from the beginning of pregnancy and this continues during the lactation months. Collado et al., [30] detected a higher number of *Bacteroides* belonging to the phylum *Bacteroidetes* in the feces of pregnant women with normal prepregnancy weight compared to overweight women.

It is remarkable how the *Bacteroidetes* and *Actinobacteria* phyla in human milk correlated directly with maternal abdominal adiposity when it was within healthy parameters, but when adiposity was higher this correlation showed an inverse trend between *Bacteroidetes* and waist circumference. In contrast, the adiposity located in the upper part of the body, represented by the bicipital and subscapular skinfolds, presented a significant direct correlation with both phyla when the skinfolds were >1 SD.

It is also interesting how the direct correlation between the *Bacteroidetes* phyla and the variables pregestational BMI ≤ 24.9, waist circumference ≤80 cm, and percentage of body fat ≤33% became inverse when exceeding the health normal limit. The *Bacteroidetes* phylum produces abundant amounts of propionate, a short chain fatty acid (SCFA) that is largely absorbed by the hepatocyte and is a good precursor of gluconeogenesis, liponeogenesis, and protein synthesis [31]. It significantly stimulates the release of peptide YY (PYY) and glucagon-like peptide-1 (GLP-1) (anorectic hormones) from human colonic cells causing a reduction in energy intake [32]. In addition, it has an effect on the decrease of abdominal adipose tissue, the content of hepatocellular lipids, and a protective effect with insulin sensitivity [33]. These findings would give a clue as to why, in our study, this phylum in human milk is abundant and correlates directly with the level of maternal adiposity when it is healthy while it decreases with overweight and obesity and is inversely associated with waist circumference.

According to our findings, milk from women with high and very high abdominal adiposity provides their infants with lower amounts of *Bacteroidetes,* which may be a predisposing factor to greater weight gain by the infants. A decrease in this bacterial phylum in the intestine of the human body has been associated with the activation of metabolic pathways. This favors the accumulation of fat in adipose tissue through the extraction of energy from indigestible carbohydrates in the diet [34,35].

The percentage of “others” was high. This is probably due to the inclusion of the phylum *Proteobacteria*, which together with the *Bacteroidetes*, *Firmicutes*, and *Actinobacteria* phyla represents approximately 98% of the bacterial phyla present in human milk. Similar findings have been observed by others [36,37,38]. As in the human intestine, the proportions of *Firmicutes* and *Bacteroidetes* had higher medians than the phylum *Actinobacteria* [3,26].

Strengths and limitations. The main strengths are that to date there are few works that have explored the bacterial phyla in human milk and the factors involved in their abundance. The limitations would be that the percentage of others could be affected in those cases in which the sterilization technique by the extraction pump carried out by the mothers had not been adequate and that variables such as gender of the baby and route of birth were not included in the multiple lineal regression. Another weakness would be that the biomolecular techniques used did not allow for determining the complete panorama of the microbiota as could be achieved by sequencing together with other biomolecular techniques.

## 5. Conclusions

The proportions of bacterial phyla in human milk were associated with maternal adiposity. The phyla *Bacteroidetes* and *Actinobacteria* showed direct and significant correlations with BMI, waist circumference, and the percentage of body fat in women with normal adiposity. There was a direct correlation between prepregnancy BMI and the proportion of *Bacteroidetes* in human milk. Therefore, the nutritional status of the woman before pregnancy and during lactation would have an important effect on the abundance of *Bacteroidetes* and *Actinobacteria* in human milk.

It is important to emphasize that this differentiated nature of the relationship of maternal adiposity with the concentration of bacterial phyla between women with normal adiposity vs. excess adiposity was observed both in the prepregnancy stage and during lactation. These results would have implications for the microbial colonization process of infants.

## Figures and Tables

**Table 1:** Bacterial phyla in human milk samples from women from the metropolitan area of Guadalajara (*n* = 70).

Variable	Median	IQR 25–75
Percentage of *Firmicutes* (%)	9.0	5.12–18.8
Percentage of *Bacteroidetes* (%)	4.4	1.9–13.8
Percentage of *Actinobacteria* (%)	2.4	1–5.4
Percentage of others (%)	73.5	56.3–86.7
F/B ratio ^1^	2.07	0.33–5.4

^1^ F—Firmicutes, B—Bacteroidetes.

**Table 2:** Anthropometric variables of nursing mothers (*n* = 70).

Variable	Median	IQR 25–75
Prepregnancy weight (kg)	62	55–70.6
Prepregnancy BMI (kg/m^2^)	24.7	21.6–27.3
Weight gain during pregnancy (kg)	12	9–14.6
Weight (kg)	64.7	57.3–73.2
Height (m)	1.60	1.5–1.6
Waist circumference (cm)	81.3	76.8–87.7
MUAC (cm)	28.3	26.5–31.8
TSF (mm)	24.5	16–29.2
SSF (mm)	21	15.7–26.5
BSF (mm)	15	11.6–22
SISF (mm)	24	14.7–30.2
BMI (Kg/m^2^)	24.9	22.8–28.6
Total body fat per BIA ^1^ (kg)	22.2	18.1–30.2
Fat percentage by BIA ^1^ (%)	34.9	30.4–38.7
Lean mass per BIA ^1^ (kg)	40.8	38–44.3
Lean mass percentage by BIA ^1^ (%)	65	61.1–69.6
Fat percentage calculated with SIRI ^2^ (%)	36.4	30.9–39.1

MUAC—medium upper arm circumference; TSF—tricipital skinfold; SSF—subscapular skinfold; BSF—bicipital skinfold; SISF—suprailiac skinfold; BMI—body mass index; ^1^ BIA—bioelectrical impedance analysis; ^2^ SIRI equation, 1993.

**Table 3:** Correlation and regression coefficients of the percentage of human milk bacterial phyla with maternal anthropometric variables in 70 nursing mothers.

Phyla (%)	*n*				r	R^2^	*p*
		Anthropometric Variables	Median	IQR (25–75)			
*Firmicutes*	24	Weight gain in pregnancy > Recommended	14.3	12–17	−0.399 ^1^	0.159	0.054
	60	BSF ≤ 1 DE	14	10–18	0.190 ^1^	0.036	0.150
*Bacteroidetes*	37	Pregestational BMI ≤ 24.9 kg/m^2^	22	19.6–23.6	0.238 ^2^	0.056	0.168
	33	Pregestational BMI ≥ 25 kg/m^2^	28	25.8–31.3	−0.402 ^1^	0.162	0.023
	36	Current BMI ≤ 24.9 kg/m^2^	22.8	21–23.9	0.375 ^2^	0.140	0.029
	27	Waist circumference ≤80 cm	75.8	70.6–78	0.433 ^1^	0.187	0.027
	43	Waist circumference >80 cm	86	81.7–91.6	−0.210 ^2^	0.044	0.187
	29	Percentage of fat ≤33%	29.8	25–31.2	0.529 ^2^	0.280	0.004
	41	Percentage of fat >33%	38.4	36.6–38.4	−0.054 ^2^	0.003	0.742
	7	TSF >1 DE	37.1	34–42	0.575 ^2^	0.330	0.177
	11	BSF >1 DE	26.5	25–34.5	0.693 ^1^	0.480	0.026
	11	SSF >1 DE	31	31–39	0.893 ^2^	0.797	<0.001
*Actinobacteria*	37	Pregestational BMI ≤ 24.9 kg/m^2^	22	19.6–23.6	−0.228 ^2^	0.052	0.188
	36	Current BMI ≤ 24.9 kg/m^2^	22.8	21–23.9	0.344 ^2^	0.118	0.047
	27	Waist circumference ≤80 cm	75.8	70.6–78	0.457 ^2^	0.208	0.016
	29	Percentage of fat ≤33 %	29.8	25–31.2	0.475 ^2^	0.226	0.011
	63	TSF ≤1 DE	23	16–28	0.252 ^1^	0.063	0.052
	59	BSF ≤1 DE	14	10–18	0.255 ^2^	0.065	0.054
	10	BSF >1 DE	26.5	25–34.5	0.820 ^2^	0.672	0.004
	56	SISF≤1 DE	19	12.3–25.8	0.303 ^2^	0.092	0.026
F/B Ratio	27	Waist circumference ≤80 cm	75.8	70.6–78	0.299 ^2^	0.089	0.139
	11	BSF >1 DE	26.5	25–34.5	0.517 ^1^	0.267	0.126
	11	SSF >1 DE	31	31–39	0.478 ^1^	0.228	0.137

BMI—body mass index; MUAC—medium upper arm circumference; TSF—tricipital skinfold; SSF—subscapular skinfold; BSF—bicipital skinfold; SISF—suprailiac skinfold; ^1^ Spearman ^2^ Pearson.

## Data Availability

The databases from which the data for this manuscript were obtained can be found at the Institute of Human Nutrition of the University of Guadalajara. Email: inhu@cucs.udg.mx; Phone +523336189667.

## Supplementary Materials

The following supporting information can be downloaded at: https://www.mdpi.com/article/10.3390/nu14142887/s1, Table S1: Quantitative variables of lactating mothers and their infants; Table S2: Qualitative variables of lactating mothers and their infants.

## References

[1] Urbaniak C., Angelini M., Gloor G.B., Reid G. (2016). Human milk microbiota profiles in relation to Birthing Method, Gestation and Infant Gender. Microbiome.

[2] Kasai C., Sugimoto K., Moritani I., Tanaka J., Oya Y., Inoue H., Tameda M., Shiraki K., Ito M., Takei Y. (2015). Comparison of the Gut Microbiota Composition between Obese and Non-obese Individuals in a Japanese Population, as Analyzed by Terminal Restriction Fragment Length Polymorphism and Next-Generation Sequencing. BMC Gastroenterol..

[3] Martin J.V., Leonard M.M., Fiechtner L., Fasano A. (2016). Transitioning from Descriptive to Mechanistic Understanding of the Microbiome: The Need for a Prospective Longitudinal Approach to Predicting Disease. J. Pediatr..

[4] Espinosa-Martos I., Jiménez E., de Andrés J., Rodríguez-Alcalá L.M., Tavárez S., Manzano S., Fernández L., Alonso E., Fontecha J., Rodríguez J. (2016). Milk and blood biomarkers associated to the clinical efficacy of a probiotic for the treatment of infectious mastitis. Benef. Microbes.

[5] Togo A., Dufour J., Lagier J., Dubourg G., Raoult D., Million M. (2019). Repertoire of human breast and milk microbiota: A systematic review. Future Microbiol..

[6] Jost T., Lacroix C., Braegger C.P., Rochat F., Chassard C. (2013). Vertical Mother-Neonate Transfer of Maternal Gut Bacteria via Breastfeeding. Environ. Microbiol..

[7] Rodriguez J.M. (2014). The Origin of Human Milk Bacteria: Is There a Bacterial Entero-Mammary Pathway during Late Pregnancy and Lactation?. Adv. Nutr..

[8] Donnet-Hughes A., Perez P.F., Doré J., Leclerc M., Levenez F., Benyacoub J., Serrant P., Segura-Roggero I., Schiffrin E.J. (2010). Potential role of the intestinal microbiota of the mother in neonatal immune education. Proc. Nutr. Soc..

[9] Osorio L.M., Umbarila A.S. (2015). Microbiota de la glándula mamaria. Pediatría.

[10] Martín R., Langa S., Reviriego C., Jiménez E., Marín M.L., Olivares M., Boza J., Jiménez J., Fernández L., Xaus J. (2004). The Commensal Microflora of Human Milk: New Perspectives for Food Bacteriotherapy and Probiotics. Trends Food Sci. Technol..

[11] Biagi E., Quercia S., Aceti A., Beghetti I., Rampelli S., Turroni S., Faldella G., Candela M., Brigidi P., Corvaglia L. (2017). The Bacterial Ecosystem of Mother’s Milk and Infant’s Mouth and Gut. Front. Microbiol..

[12] Boix-Amorós A., Collado M.C., Mira A. (2016). Relationship between Milk Microbiota, Bacterial Load, Macronutrients, and Human Cells during Lactation. Front. Microbiol..

[13] Cabrera-Rubio R., Mira-Pascual L., Mira A., Collado M.C. (2015). Impact of mode of delivery on the milk microbiota composition of healthy women. J. Dev. Orig. Health Dis..

[14] Avershina E., Angell I.L., Simpson M., Storrø O., Øien T., Johnsen R., Rudi K. (2018). Low Maternal Microbiota Sharing across Gut, Breast Milk and Vagina, as Revealed by 16S rRNA Gene and Reduced Metagenomic Sequencing. Genes.

[15] Monks J., Orlicky D., Stefanski A.L., Libby A.E., Bales E.S., Rudolph M.C., Johnson G.C., Sherk V.D., Jackman M.R., Williamson K. (2018). Maternal obesity during lactation may protect offspring from high fat diet-induced metabolic dysfunction. Nutr. Diabetes.

[16] Gaillard R. (2015). Maternal obesity during pregnancy and cardiovascular development and disease in the offspring. Eur. J. Epidemiol..

[17] Gordon-Larsen P., Adair L.S., Suchindran C.M. (2007). Maternal obesity is associated with younger age at obesity onset in U.S. adolescent offspring followed into adulthood. Obesity.

[18] Collado M.C., Laitinen K., Salminen S., Isolauri E. (2012). Maternal Weight and Excessive Weight Gain during Pregnancy Modify the Immunomodulatory Potential of Breast Milk. Pediatr. Res..

[19] Garcia-Mantrana I., Collado M.C. (2016). Obesity and overweight: Impact on maternal and milk microbiome and their role for infant health and nutrition. Mol. Nutr. Food Res..

[20] Murphy K., Curley D., O’Callaghan T.F., O’Shea C., Dempsey E.M., O’Toole P.W., Ross R.P., Ryan C.A., Stanton C. (2017). The Composition of Human Milk and Infant Faecal Microbiota over the First Three Months of Life: A Pilot Study. Sci. Rep..

[21] Diario Oficial de la Federación (2013). NORMA Oficial Mexicana NOM-043-SSA2-2012, Servicios Básicos de Salud. Promoción y Educación Para la Salud en Materia Alimentaria. Criterios Para Brindar Orientación. https://www.dof.gob.mx/nota_detalle.php?codigo=5285372&fecha=22/01/2013#gsc.tab=0.

[22] Marfell-Jones M.J., Stewart A.D., de Ridder J.H. (2011). International Standards for Anthropometric Assessment.

[23] Rasmussen K.M., Yaktine E.L. (2009). Institute of Medicine (US) and National Research Council (US) Committee to Reexamine IOM Pregnancy Weight Guidelines; The National Academies Collection—Reports funded by National Institutes of Health. Weight Gain during Pregnancy: Reexamining the Guidelines.

[24] Gallagher D., Heymsfield S., Heo M., Jebb S.A., Murgatroyd P.R., Sakamoto Y. (2000). Healthy percentage body fat ranges: An approach for developing guidelines based on body mass index. Am. J. Clin. Nutr..

[25] Siri W. (1993). Body Composition from Fluid Spaces and Density: Analysis of Methods. 1961. Nutrition.

[26] Koliada A., Syzenko G., Moseiko V., Budovska L., Puchkov K., Perederiy V., Gavalko Y., Dorofeyev A., Romanenko M., Tkach S. (2017). Association between body mass index and *Firmicutes/Bacteroidetes* ratio in an adult Ukrainian population. BMC Microbiol..

[27] Kumar H., du Toit E., Kulkarni A., Aakko J., Linderborg K.M., Zhang Y., Nicol M.P., Isolauri E., Yang B., Collado M.C. (2016). Distinct Patterns in Human Milk Microbiota and Fatty Acid Profiles across Specific Geographic Locations. Front. Microbiol..

[28] Eckburg P.B., Bik E.M., Bernstein C.N., Purdom E., Dethlesfsen L., Sargent M., Gill S.R., Nelson K.E., Relman D.A. (2005). Diversity of the Human Intestinal Microbial Flora. Science.

[29] Riva A., Borgo F., Lassandro C., Verduci E., Morace G., Borghi E., Berry D. (2017). Pediatric obesity is associated with an altered gut microbiota and discordant shifts in *Firmicutes* populations. Environ. Microbiol..

[30] Collado M.C., Isolauri E., Laitinen K., Salminen S. (2008). Distinct Composition of Gut Microbiota during Pregnancy in Overweight and Normal-Weight Women. Am. J. Clin. Nutr..

[31] den-Besten G., van Eunen K., Groen A.K., Venema K., Reijngoud D., Bakker B.M. (2013). The role of short-chain fatty acids in the interplay between diet, gut microbiota, and host energy metabolism. J. Lipid Res..

[32] Müller T.D., Finan B., Bloom S.R., D’Alessio D., Drucker D.J., Flatt P.R., Fritsche A., Gribble F., Grill H.J., Habener J.F. (2019). Glucagon-like peptide 1 (GLP-1). Mol. Metab..

[33] Machate D.J., Figueiredo P.S., Marcelino G., Avellaneda-Guimarães R.C., Hiane P.A., Bogo D., Zorgetto-Pinheiro V.A., Silva-de Oliveira L.C., Pott A. (2020). Fatty Acid Diets: Regulation of Gut Microbiota Composition and Obesity and Its Related Metabolic Dysbiosis. Int. J. Mol. Sci..

[34] Rial S.A., Karelis A.D., Bergeron K., Mounier C. (2016). Gut Microbiota and Metabolic Health: The Potential Beneficial Effects of a Medium Chain Triglyceride Diet in Obese Individuals. Nutrients.

[35] Indian C., Rizzardi K., Castelo P., Ferraz L., Darrieux M., Parisotto T. (2018). Childhood Obesity and *Firmicutes/Bacteroidetes* Ratio in the Gut Microbiota: A Systematic Review. Child Obes..

[36] Lackey K.A., Williams J.E., Meehan C.L., Zachek J.A., Benda E.D., Price W.J., Foster J.A., Sellen D.W., Kamau-Mbuthia E.W., Kamundia E.W. (2019). What’s Normal? Microbiomes in Human Milk and Infant Feces Are Related to Each Other but Vary Geographically: The INSPIRE Study. Front. Nutr..

[37] Marín-Gómez W., Grande M.J., Pérez-Pulido R., Galvez A., Lucas R. (2020). Changes in the Bacterial Diversity of Human Milk during Late Lactation Period (Weeks 21 to 48). Foods.

[38] Ruiz L., Alba C., García-Carral C., Jiménez E.A., Lackey K.A., McGuire M.K., Meehan C.L., Foster J., Sellen D.W., Kamau-Mbuthia E.W. (2021). Comparison of Two Approaches for the Metataxonomic Analysis of the Human Milk Microbiome. Front. Cell. Infect. Microbiol..

[39] Diario Oficial de la Federación (2002). NORMA Oficial Mexicana NOM-087-ECOL-SSA1-2002, Protección Ambiental-Salud Ambiental-Residuos Peligrosos Biológicoinfecciosos—Clasificación y Especificaciones de Manejo. https://www.cndh.org.mx/DocTR/2016/JUR/A70/01/JUR-20170331-NOR14.pdf.

