# Supplementary material for: Firmicutes, Bacteroidetes and Actinobacteria in Human Milk and Maternal Adiposity
Source: Nutrients. 2022 Jul 14;14(14):2887. doi: 10.3390/nu14142887 (PMC9315738; doi:10.3390/nu14142887)
Supplement: Supplementary file 1 [file nutrients-14-02887-s001.zip › nutrients-1780731-supplementary.pdf]

Supplementary Table S1. Quantitative variables of lactating mothers and their infants (*n* = 70).

Variable	Median	IQR (25-75)	Min and max
Age (years)	30	25-32	17-40
Pregnancy number	1	1-2	1-6
Infant's age (weeks)	10.50	6-15	4-18

Supplementary Table S2. Qualitative variables of lactating mothers and their infants (*n* = 70).

	Variable	Frecuency	(%)
**Marital Status**	Single	9	12.9
	Merried	50	71.4
	Unmarried cohabitation	11	15.7
**Scholarship**	Elementary School	1	1.4
	Secundary School	2	2.9
	Undergraduate high school	2	2.9
	High school	9	12.9
	Specialized professional technician	2	2.9
	Incomplete bachelor`s degree	9	12.9
	Bachelor´s degree	25	35.7
	Posgraduate	18	25.7
**Occupation**	Student	6	8.6
	House wife	21	30
	Employee	3	4.3
	Business woman	3	4.3
	Profesional	35	50
	Other	2	2.9
**Type of delivery**	Vaginal delivery	26	37.1
	Caesarean section	44	62.9
**Gender of the infant**	Famale	25	35.7
	Male	45	64.3
**Type of breastfeeding ***	Exclusive breastfeeding	50	71.4
	Partial breastfeeding	20	28.6

* Vásquez-Garibay, 2016.
